# Surgical vs. Conservative Management for Lobar Intracerebral Hemorrhage, a Meta-Analysis of Randomized Controlled Trials

**DOI:** 10.3389/fneur.2021.742959

**Published:** 2022-01-20

**Authors:** Muhammad Junaid Akram, Rui Zhao, Xue Shen, Wen-Song Yang, Lan Deng, Zuo-Qiao Li, Xiao Hu, Li-Bo Zhao, Peng Xie, Qi Li

**Affiliations:** ^1^Department of Neurology, The First Affiliated Hospital of Chongqing Medical University, Chongqing, China; ^2^Department of Neurology, Yongchuan Hospital of Chongqing Medical University, Chongqing, China; ^3^National Health Commission Key Laboratory of Diagnosis and Treatment on Brain Functional Diseases, The First Affiliated Hospital of Chongqing Medical University, Chongqing, China

**Keywords:** intracerebral hemorrhage, surgical management, conservative management, randomized controlled trial, meta-analysis

## Abstract

**Background:**

Outcomes regarding the conventional surgical and conservative treatment for the lobar intracerebral hemorrhage (ICH) have not been previously compared. The current meta-analysis was designed to review and compile the evidence regarding the management of patients with lobar intracerebral hemorrhage.

**Methods:**

Online electronic databases, including PubMed, Embase, Medline, Cochrane Library, and Google Scholar, were searched for randomized controlled trials (RCTs). Studies were selected on the basis of the inclusion and exclusion criteria. Trials with CT-confirmed lobar intracerebral hemorrhage patients of which treatment regimen was started within 72 h following the stroke were included. Low quality trials were excluded. Death or dependence was defined as primary outcome and death at the end of the follow up was the secondary outcome.

**Results:**

One hundred five RCTs were screened and 96 articles were excluded on the basis of abstract. Nine articles were assessed for the eligibility and 7 trials were included that involved 1,102 patients. The Odds ratio (OR) for the primary outcome was 0.80 (95% CI, 0.62–1.04, *p* = 0.09) and for the secondary outcome was 0.79 (95%CI, 0.60–1.03, *p* = 0.09).

**Conclusion:**

Our findings suggested that surgical treatments did not significantly improve the functional outcome as compared with the conservative medical management for patients with lobar ICH.

## Introduction

Stroke is a major public health concern contributing 10% to all deaths worldwide and 5% loss to disability-adjusted life-years (1). Stroke accounts for high levels of morbidity and mortality even in treated patients (2). Intracerebral hemorrhage (ICH) causes more loss to disability-adjusted life-years than ischemic stroke (3). Stroke is the second most prevalent cause of death in China, contributing one-third of total deaths worldwide (4, 5). As one of the fatal types of stroke, spontaneous intracerebral hemorrhage (ICH) has an incidence of 24.6 per 100,000 person-years and mortality rate of 40% at 1 month in adults (6). The incidence of ICH varies among populations (7). Cerebral amyloid angiopathy (CAA) associated vasculopathies lead to lobar intracerebral hemorrhage—a sub-type of intracranial hemorrhage (8). CAA is the second most common cause leading to ICH following hypertension. The incidence of CAA-related lobar ICH in elderly has been increasing (8–10). About two-third of cases of spontaneous ICH are deep ICH, and lobar ICH accounts for the remaining one-third. Lobar ICH involves the cortical and subcortical areas, and follows a lobar pattern across one or multiple brain lobes (10). The rate of recurrence in lobar hemorrhagic patients is as high as 4% per patient-year (11).

The management techniques for ICH have remained controversial. Many studies have compared the surgical procedures, including minimally invasive surgery, endoscopic surgery, stereotactic aspiration, keyhole surgery, craniotomy, and craniopuncture with conservative medical management for ICH (12). Wang et al. reported that minimally invasive surgery (MIS) had improved functional outcomes compared to the conservative medical management for patients with intracerebral hemorrhage (13). Contrary to the above findings, the results of two research studies have shown that MIS had no advantage over medical management (14, 15). Moreover, another study explored that MIS procedure had significantly better results for ICH than other procedures like open surgery and conservative medical management (16). Minimally invasive procedures have evolved into different novel surgical techniques for Intracerebral hemorrhage (13, 16–19).

Image guided MIS plus alteplase in intracerebral hemorrhage evacuation (MISTIE II) procedure seems safe in patients with ICH, but with cautionary findings of increased asymptomatic bleeding (20). MISTIE III trials showed that MIS procedure was safe in patients with ICH, but showed no improvement in the functional outcome for moderate to large ICH compared to standard medical care (21).

In recent years, many treatment techniques, including stereotactic aspiration, MIS, endoscopic surgery, and craniotomy have been widely used for ICH treatment. The purpose of this study was to pool all the randomized controlled trials (RCTs) determining the effects of surgical and conservative management for the patients with lobar ICH. The literature does not highlight any definite technique, and explicitly focuses on lobar intracerebral hemorrhage. These results may help clinicians to choose evidence-based treatment for lobar ICH.

## Methods

### Data Extraction and Search Strategy

A literature search was performed on the electronic databases, including PubMed, Embase, Medline, Cochrane Library, and Google Scholar from 1980 to 2020. The combination of the following keywords was used to locate the related research articles: “surgery” or “craniotomy” or “minimally invasive procedure” or “endoscopic” and “conservative” or “medical management” or “non-surgical” and “lobar hemorrhage” or “intracerebral hemorrhage” or “supratentorial” or “subcortical” or “hematoma.” Searched studies were selected based on the inclusion and exclusion criteria (Supplementary Table 1).

### Eligibility Criteria

The inclusion criteria for the studies were as follows: (1) CT- or MRI-confirmed diagnosis of patients with lobar intracerebral hemorrhage, (2) treatment regimen of the patients was started within 72 h following the stroke, (3) RCTs comparing the surgical treatment with the conservative medical management, and (4) age ≥ 18 years. Exclusion criteria were as follows: (1) intracerebral hemorrhage caused by ruptured aneurysms, arteriovenous malformation (AVM), vascular anomaly, brain tumors, traumatic brain injury, or coagulopathy; and (2) studies who included patients with infratentorial hemorrhage.

### Types of Intervention

The interventions used for the treatment purpose comprised the surgical treatment (endoscopic surgery, open craniotomy, stereotactic aspiration, and endoscopic surgery + stereotactic aspiration) and conservative management (pharmacological, non-surgical). Decision regarding the inclusion of the studies was made independently by the authors.

### Main Measurements Examined

Reported data showed that studies utilized different outcome measures in order to determine the results. The mainly used outcome measures were Glasgow Outcome Scale (GOS), The Barthel Index, and The Modified Rankin Scale (14, 22–27). An unfavorable outcome was considered as a primary outcome in the current study. Vegetative state or death and severe disability according to the GOS reflected unfavorable outcomes. In the absence of the GOS, the modified Rankin scale equal to or greater than 3 (≥3) or a Barthel index score of 90 represented the unfavorable outcome. Whereas, in the Surgical Treatment for Ischemic Heart Failure (STICH) and STITCH II studies, Extended Glasgow Outcome Scale (GOSE) was used to determine the level of independence of the patients (25, 26). Being independent in performing activities outside the home was considered a favorable outcome. The prognostic score for these studies was calculated by the “10 × GCS – Age - 0.64 × Volume,” giving a cutoff value of 27.672 to divide the outcome in favorable and unfavorable.

### Quality and Risk of Bias Assessment

Quality of the studies was assessed using the Cochrane criteria, comprising four aspects, namely, (1) random sequence generation, (2) allocation concealment, (3) blinding of outcome assessment, and (4) incomplete outcome data reported. The first three points were scored as “No = 0, Unclear = 1, Yes = 2” and the fourth was scored as “No = 0, Yes = 1.” All the studies with a score >2 were included in the data synthesis and the rest were regarded as low-quality studies. All the studies were sorted based upon inclusion and exclusion criteria and assessed for quality by the authors. Statistical analysis was performed using RevMan5 Software. Fixed effects meta-analysis was used to pool the events rate across the studies. Funnel plot was used to indicate the presence of specific publication bias (Supplementary Figure 1). *I*^2^ statistics was used to determine the heterogeneity of the studies. Risk of bias summary of the study was analyzed (Supplementary Figure 2).

## Results

### Study Selection

**Figure 1:**
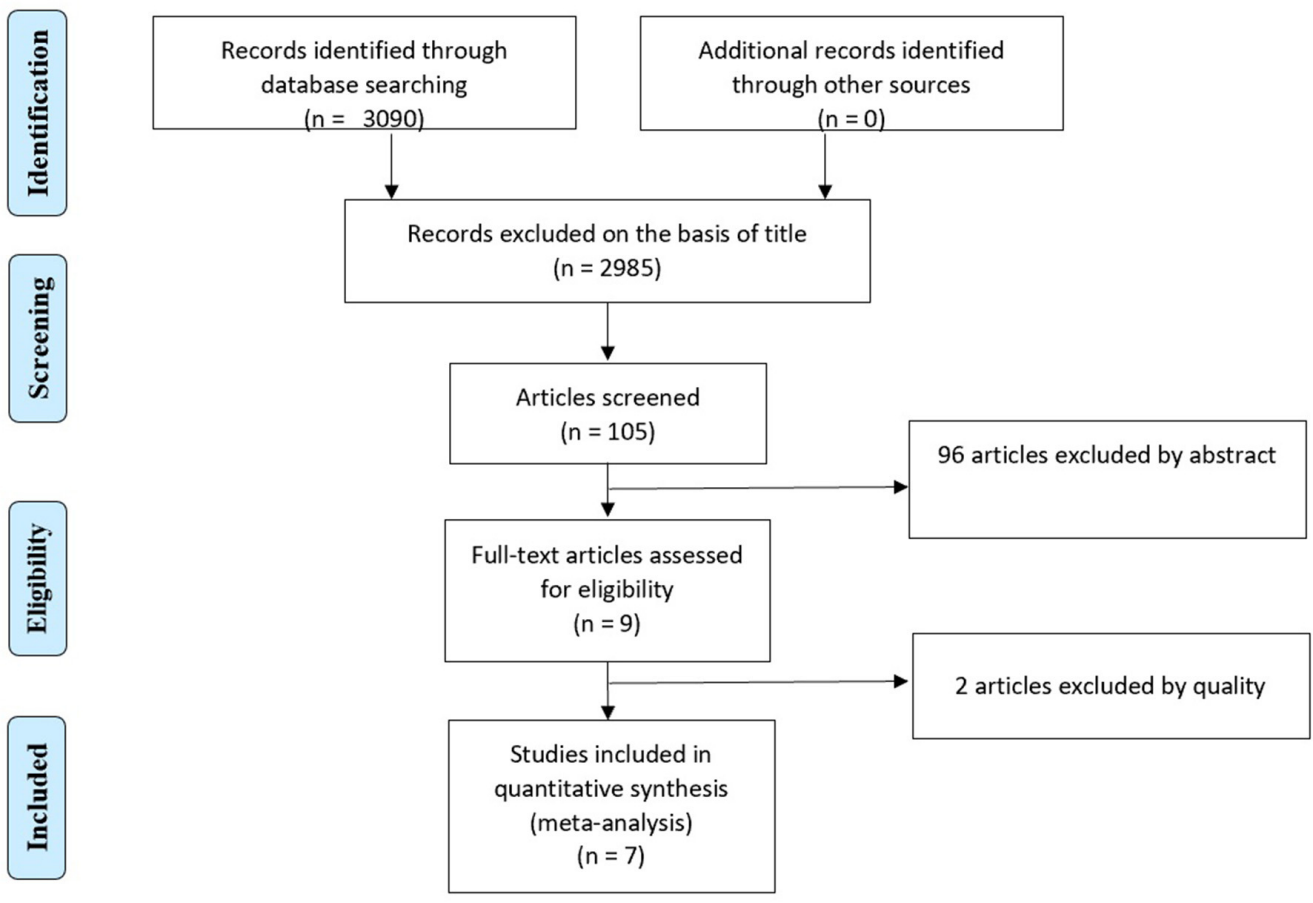
PRISMA Flow chart of study selection.

There were 3,090 studies initially retrieved. Two thousand nine hundred eighty-five studies were excluded on the basis of dissimilarity in the title. The remaining 105 studies were screened in total, out of which, 96 studies were excluded on the basis of abstract. Nine remaining articles were assessed for the quality based upon the Cochrane eligibility criteria. As a cut off score of <2 was a set point value for the low-quality study, 2 studies were excluded on quality basis. Thus, seven trials were considered eligible for the inclusion in the study (Figure 1).

### Main Outcomes Description

Among seven studies comparing the surgical treatment with the conservative management for lobar ICH, three studies involved endoscopic surgery and stereotactic aspiration, two studies involved the open craniotomy, one study endoscopic surgery, and another study involved only stereotactic aspiration. The other treatment group in seven studies received the conservative medical management.

**Figure 2:**
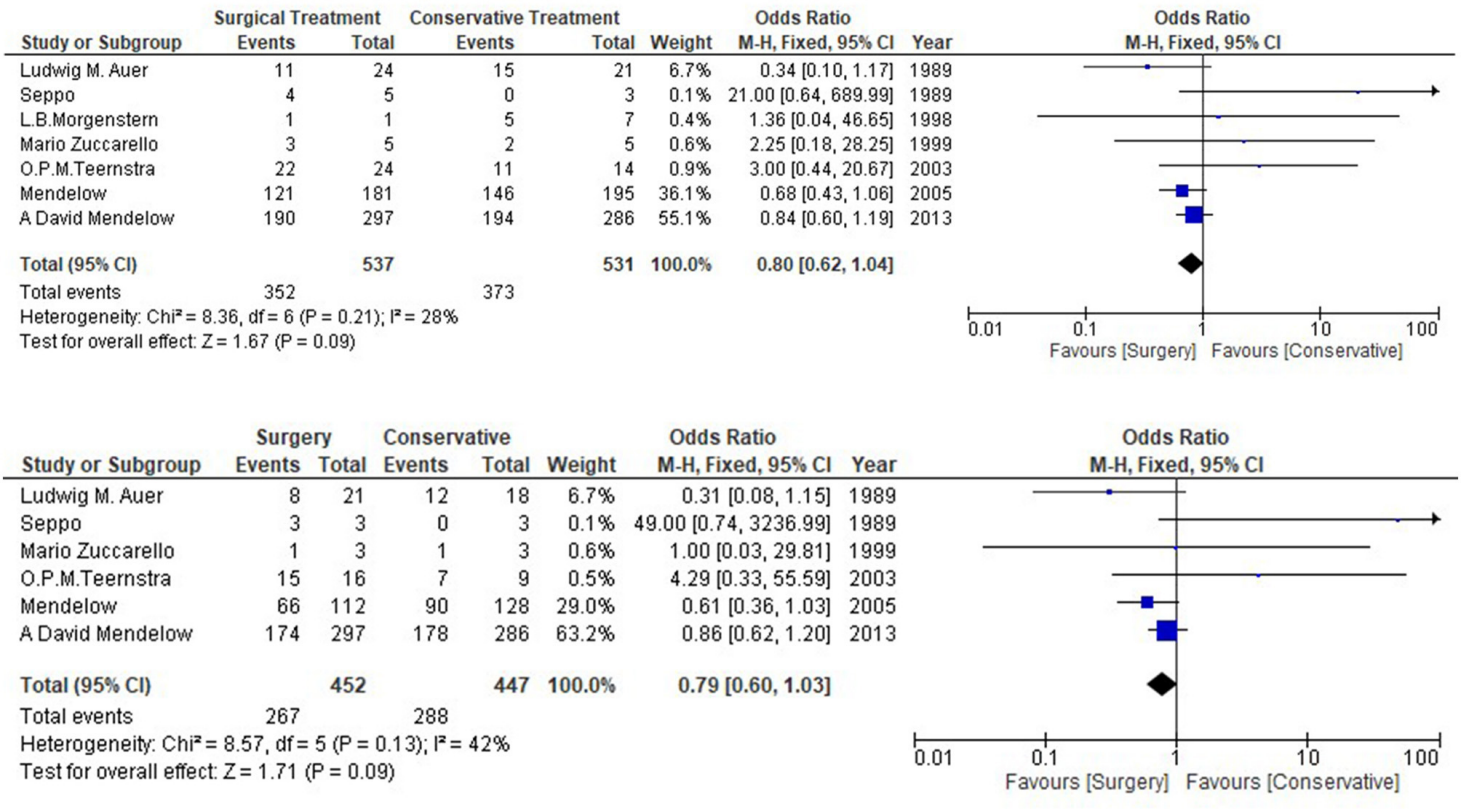
Overall analysis of surgical vs. conservative medical management.

Table 1 shows the details of included studies, treatment protocols, timing of the surgery, and the quality of the studies. The clinical characteristics and outcomes of included studies are described in Table 2. The total number of the patients with lobar ICH was 1,102. Among them, 552 were in the surgical group and 550 in the conservative group. All the studies provided data regarding death or dependence at the end of the follow up (Figure 2). Mendelow et al. included the maximum number of the patients as compared to other studies. The overall results showed a non-significant trend toward better prognosis in the surgical group (OR 0.80, 95% CI 0.62–1.04; *p* = 0.09).

**Table 1:** Baseline summary of included studies.

**Trials**	**Treatments**		**Surgery timings**	**Quality of literature**				
				**Randomized generation**	**Outcome blinding**	**Incomplete data**	**Allocation concealment**	**Total**
Ludwig M. Auer	Endoscopic surgery	Conservative medical	<48	1	0	1	0	2
Seppo Juvela	Open craniotomy	Conservative medical	<24	2	0	1	0	3
L. B.Morgenstern	Open craniotomy	Conservative medical	<12	2	1	1	2	6
Zuccarello	Stereotactic aspiration	Conservative	<24	2	0	1	2	5
O. P. M.Teernstra	Stereotactic aspiration	Conservative medical	<72	2	0	1	2	5
Mendelow	Endoscopic surgery + Stereotactic aspiration	Conservative medical	<24	2	2	1	2	7
A. David Mendelow	Endoscopic surgery + Stereotactic aspiration	Conservative medical	<48	2	2	1	2	7

**Table 2:** Main characteristics and outcomes of included studies.

**References**	**No. of ICH cases**	**SG**	**CG**	**Lobar SG**	**Lobar CG**	**Total lobar cases**	**Lobar hematoma %**	**Primary** **outcome**	**Secondary** **outcome**
Auer et al. (14)	100	50	50	24	21	45	45	6 mo. Outcome (11/24:15/21)	6 mo. Death (8/21:12/18)
Juvela et al. (22)	52	26	26	5	3	8	15	6 mo. Outcome (4/5:0/3)	6 mo. Death (3/3:0/3)
Morgenstern et al. (27)	34	17	17	1	7	8	24	6 mo. Outcome (1/1:5/7)	NR
Zuccarello et al. (23)	20	9	11	5	5	10	50	3 mo.BI (3/5:2/5)	3 mo. Death (1/3:1/3)
Teernstra et al. (24)	70	36	34	24	14	38	54	6 mo. mRS (22/24:11/14)	6 mo. Death (15/16:7/9)
Mendelow et al. (25)	1,033	503	530	196	214	410	40	6 mo. BI (121/181:146/195)	6 mo. Death (66/112:90/128)
Mendelow et al. (26)	601	307	294	297	286	583	97	6 mo. Outcome (190/297:194/286)	6 mo. death (174/297:178/286)
Total	1,910	948	962	552	550	1,102	58		

*SG, surgical group; CG, conservative group; NR, not reported*.

Secondary outcome was recorded for 410 patients with lobar intracerebral hemorrhage. Auer and Mendelow contributed the most cases of the patients, 45 and 38, respectively, to the meta-analysis. No significant difference (OR 0.79, 95% CI 0.60–1.03, *p* = 0.09) was observed in the secondary outcome between surgical and conservative medical management group.

## Discussion

The current meta-analysis included seven articles without any conflict or controversial findings. It was intended to determine the best possible effects of two treatments, surgical and conservative, for lobar patients with ICH. The management for intracerebral hemorrhage has remained controversial and depends greatly on the patient and baseline clinical characteristics, including GCS score, volume, and location of the hematoma. The results of current meta-analysis were consistent with the included researches and showed no significant differences between surgical and conservative medical management of the patients with lobar ICH.

A limited number of randomized controlled trials have focused on the management of lobar intracerebral hemorrhage. Mendelow et al. performed the trials focusing the lobar intracerebral hemorrhage management. STICH AND STITCH II have demonstrated the treatment priorities, considering the surgical or conservative treatments in detail, and the studies stated no significant differences between the percentage of favorable outcomes in the conservative and medical management groups (25, 26). The remaining data included in our study is extracted from literature that included other sub-types of hematoma, including thalamic, basal ganglia, and putaminal.

Patients involving only lobar hematoma were considered and outcomes were analyzed for patients with lobar intracerebral hemorrhage (14, 22–24, 27). Moreover, the specific data, including GCS score, volume of hematoma, and age of the patients, was lacking for the patients with lobar intracerebral hemorrhage. Most of the studies have described overall GCS scores and hematoma volume for intracerebral hemorrhage (14, 22, 27). Surgical evacuation failure in ICH has attributed to the high levels of morbidity related to surgical techniques (28).

The current meta-analysis showed lower heterogeneity (*p* = 0.09, *I*^2^ = 28%) for the primary outcome and it was higher for the secondary outcome (*p* = 0.09, *I*^2^ = 42%) (Figure 2). Prognosis-based outcome analysis indicated that there was no significant evidence that supports surgical method has better outcome comparing with conservative medical management in patients with lobar intracerebral hemorrhage. STITCH II trial has suggested that conscious patients with lobar hematomas have greater survival advantage when the prognosis is poor (Glasgow Coma Scale Score 9-12) and patients are assigned randomly within 21 h (26). This marginal benefit is lost when the patients have better prognosis because they are mostly operated when the condition is deteriorated. RCTs, except the STITCH II trials, have not reported the specific GCS score for lobar intracerebral hemorrhagic patients. Thus, the results can differ if the GCS is known for the other studies.

The results may also change if open surgery and minimally invasive surgery are separately considered, but the lack of effect of surgical treatment is the consequence of surgery being beneficial in some patients while not in others. Moreover, the minimally invasive techniques may be beneficial for intraventricular hemorrhages and deep clots, which require more trials.

Other surgical approaches, including craniectomy and minimally invasive surgery with thrombolysis in ICH evacuation comparing with the conservative management for the lobar intracerebral hemorrhage, should be investigated. The included studies have used different statistical approaches so the analysis have been done with caution.

## Conclusion

Our study suggested that there was no significant difference between the surgical and the conservative medical treatment for patients with lobar ICH. Future trials with larger sample sizes and standardized procedures are needed to determine the treatment effect of minimal invasive surgery in lobar ICH.

## Data Availability Statement

The original contributions presented in the study are included in the article/Supplementary Material, further inquiries can be directed to the corresponding author/s.

## Author Contributions

QL and MA contributed to the study concept and design. MA contributed in the statistical analysis. MA, RZ, and QL contributed to the drafting of the manuscript. QL, MA, PX, and L-BZ contributed to the critical revision of the manuscript for important intellectual content. QL obtained funding. QL, PX, and L-BZ contributed to the study supervision. All authors contributed to the acquisition of data, analysis, and interpretation of data.

## Funding

This study was supported by grants from the National Natural Science Foundation of China (No. 82071337), the National Key R&D Program of China (Nos. 2018YFC1312200 and 2018YFC1312203), the Foundation and Frontier Program of Chongqing Science and Technology Commission (No. cstc2018jcyjAX0341), the Chongqing Key Program of Technological Innovation and Application Development (No. cstc2019jscx-gksbX0064), the Public Welfare Science and Technology Projects of Yongchuan District in 2019 (No. Ycstc,2019nb02029), the Public Welfare Science and Technology Projects of Yongchuan District in 2018 (No. Ycstc,2018nb0223).

## Conflict of Interest

The authors declare that the research was conducted in the absence of any commercial or financial relationships that could be construed as a potential conflict of interest.

## Publisher's Note

All claims expressed in this article are solely those of the authors and do not necessarily represent those of their affiliated organizations, or those of the publisher, the editors and the reviewers. Any product that may be evaluated in this article, or claim that may be made by its manufacturer, is not guaranteed or endorsed by the publisher.
