# Supplementary material for: Surgical vs. Conservative Management for Lobar Intracerebral Hemorrhage, a Meta-Analysis of Randomized Controlled Trials
Source: Front Neurol. 2022 Jan 20;12:742959. doi: 10.3389/fneur.2021.742959 (PMC8810825; doi:10.3389/fneur.2021.742959)

**Supplement Figure 1**

Funnel Plot showing publication bias analysis results


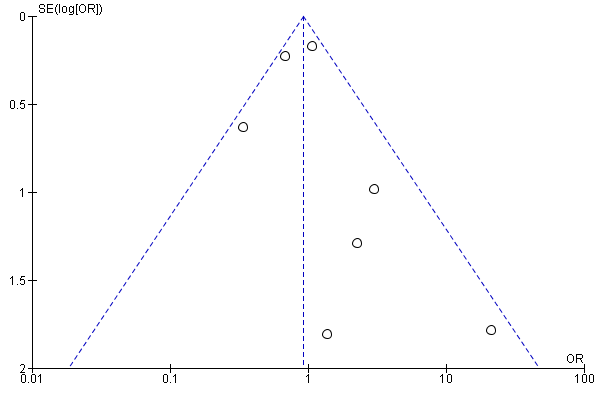


**Supplement table 1**

Literature screening strategy

| PubMed / Google Scholar/Cochrane Library/Medline | The combination of the following keywords was used to locate the related research articles: “Surgery” or “craniotomy” or “minimally invasive procedure” or “endoscopic” and “conservative” or “medical management” or “non-surgical” and “lobar hemorrhage” or “Intracerebral hemorrhage” or “Supratentorial” or “Subcortical” or “hematoma”.  **Filters : Randomized controlled Trials** |
| --- | --- |

**Supplementary Figure 2**

Risk of bias Summary


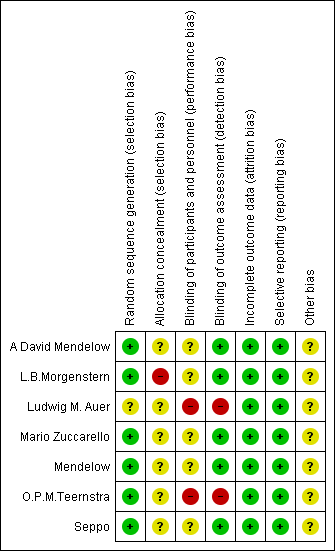

Supplement: Supplementary file 1 [file Table_1.DOCX]
